# A Review of fMRI Simulation Studies

**DOI:** 10.1371/journal.pone.0101953

**Published:** 2014-07-21

**Authors:** Marijke Welvaert, Yves Rosseel

**Affiliations:** Department of Data Analysis, Ghent University, Gent, Belgium; University Of Cambridge, United Kingdom

## Abstract

Simulation studies that validate statistical techniques for fMRI data are challenging due to the complexity of the data. Therefore, it is not surprising that no common data generating process is available (i.e. several models can be found to model BOLD activation and noise). Based on a literature search, a database of simulation studies was compiled. The information in this database was analysed and critically evaluated focusing on the parameters in the simulation design, the adopted model to generate fMRI data, and on how the simulation studies are reported. Our literature analysis demonstrates that many fMRI simulation studies do not report a thorough experimental design and almost consistently ignore crucial knowledge on how fMRI data are acquired. Advice is provided on how the quality of fMRI simulation studies can be improved.

## Introduction

Twenty years ago, functional magnetic resonance imaging (fMRI) was established as a method to measure brain activity [1], [2]. In these past twenty years, this technique has been used increasingly and has pioneered the search to map and connect the brain that has caused a world-wide collaboration of scientists from different disciplines. Engineers and physicists are intrigued by the acquisition of the fMRI data, while physicians and psychologists are challenged to adapt their behavioural experimental protocols to the scanner environment. Last but not least, the analysis of fMRI data has been, and still is, a topic of numerous discussions among statisticians. The latter are confronted with the fact that the data acquired through fMRI have no ground truth. This ground truth is needed to ensure validation of the statistical methods that are used to analyse the data and to assess statistical properties such as sensitivity, specificity, bias and robustness. Great efforts to establish this ground truth have gone into the development of mechanical models [3], while direct measuring of the neural activity with intracranial EEG (iEEG) offers another solution [4]. However, for most studies iEEG may not be feasible and simulations may be the only realistic approach to establish the ground truth of fMRI data.

NeuroImage, one of the flagship journals in the neuroimaging community, celebrated the 20th anniversary of the first fMRI publications with a special volume that consisted of 103 reviews about the early beginnings, developments in acquisition, software, processing and methodology, and prospectives for the future of fMRI [5]. Although the advances in statistical methods for fMRI data are discussed in several of these reviews, simulations *an sich* are not mentioned. In general, it appears that simulation studies are still not standard practice for fMRI methods validation. A possible explanation is that it can be quite challenging to simulate fMRI data. Not only is the coupling between the neural activity and the Blood Oxygenation Dependent Level (BOLD) not completely understood [6], fMRI data are also characterised by a great deal of noise coming from multiple sources [7]. Consequently, no common data generating process for fMRI data is available and the data generation in fMRI simulation studies is mostly defined *ad hoc*.

The goal of this review is to provide an overview of the most common data generation methods used in fMRI simulation studies. An established and accepted data generating process does not yet exist and therefore an investigation of the existing published models is called for. In particular, the validity of these data generating methods is analysed and the overall reporting and conduct of fMRI simulation studies is critically reviewed. The rest of the paper is organised as follows: In the Methods section the article selection criteria are reported that were applied to establish a database of fMRI simulation studies literature, and the focus points of the article evaluation are discussed. The Results section focuses on different aspects of the simulation studies, namely, the goals of the studies, the experimental design under investigation, the simulation parameters and the data generation models. Finally, in the Discussion, best practice recommendations are provided to increase the reliability and generalisability of fMRI simulation studies.

## Materials and Methods

### Article selection

**Figure 1:**
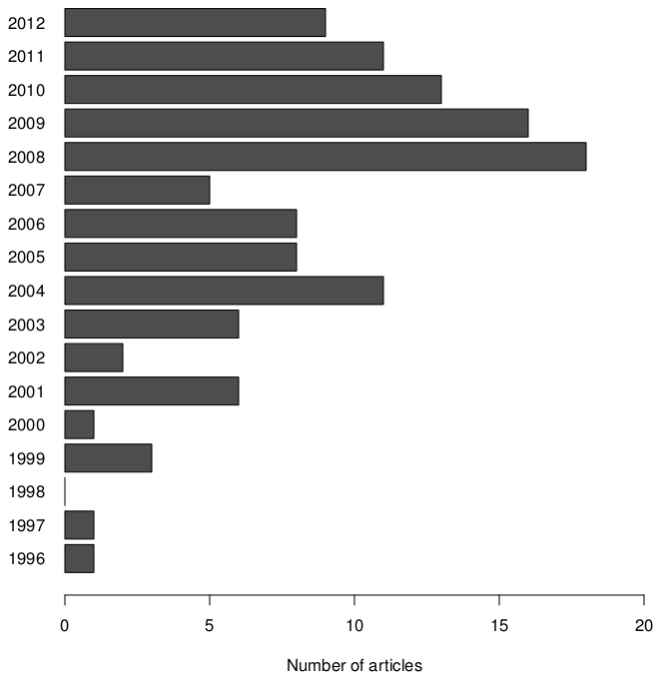
Overview of number of articles for each publication year included in the survey.

Articles were selected from the Web of Science database using the following query: “*fmri* AND *simulation* AND (*statistics* OR *data analysis*)”. By excluding articles labelled as reviews or proceedings, this search resulted in 318 hits (Result as of January, 1st 2013). All these articles were manually inspected on content and relevance. This screening resulted in excluding articles based on the following criteria: the conducted simulations were for another modality (e.g. PET, EEG, MEG, …); no time series were simulated (e.g. inference methods are often validated on simulated statistical maps); non-human fMRI was simulated; and no simulation study was conducted (e.g. papers presenting simulation software). After exclusion, the remaining 119 articles were taken into account in this analysis. Full bibliographic details of our sample can be found in the Supporting Information (Table S1). These articles were published in 39 peer-reviewed academic journals (Table 1) over a period of 16 years (Figure 1). In this sample, most simulation studies were published in NeuroImage (37), Human Brain Mapping (11), IEEE Transactions on Medical Imaging (10), Magnetic Resonance Imaging (7), IEEE Transaction on Biomedical Engineering (6) and the Journal of Magnetic Resonance Imaging (6).

**Table 1:** Overview of journals in the survey. Full details of the included studies can be found in the supporting information (Table S1).

Journal title	Number of articles
NeuroImage	37
Human Brain Mapping	11
IEEE Transactions on Medical Imaging	10
Magnetic Resonance Imaging	7
IEEE Transactions on Biomedical Engineering	6
Journal of Magnetic Resonance Imaging	6
Magnetic Resonance in Medicine	4
Other	38

During our article selection, we focused on simulation studies conducted to validate or compare analysis procedures for BOLD-fMRI data. In order to perform this validation, a data generating process results in artificial data that reflect to some degree the characteristics of real measured fMRI data. From a statistical perspective, scanning parameters that influence magnetic properties of the data (e.g. flip angle) are of less importance since they mainly have an effect on the signal-to-noise ratio. For instance, when these scanning parameters are optimised, the baseline signal might increase while the noise level decreases. The crucial aspect is to determine the components in the data that are expected to have an effect on the data analysis and model these components while generating the simulated fMRI data.

### Article evaluation

In the present study, we analysed the sections describing the simulation study for the selected papers. Where necessary the appendices or supplementary materials were also included and whenever there was still missing information after screening these sections, the whole paper was searched for this information. Only the reported methodology was evaluated (i.e. no authors were contacted for more information). There might be a discrepancy between the conducted and reported simulation studies (e.g. not all details are mentioned), however, to ensure reproducible science all critical elements should be reported. It may not always be feasible to report everything in the main text, but academic journals allow for crucial content to be described in appendices or through online supplementary materials. For each study we evaluated the goal of the simulation study, the simulation parameters and the data generating process. In the case that multiple simulation studies were present in the article, this information was retrieved from the most complex case that was described. In the Results section, summarised results are presented. For a detailed results list on the individual study level, the reader is referred to Table 2.

**Table 2:** Detailed results for the analysis of the fMRI simulation database, the ID numbers refer to the references in Table S1.

ID	Auth.	Journal	Year	Model	Design	dim.	nS	rep	parV	parJ	HRFm	HRFv	type	Noise model	Noise corr.
1	Afshin-Pour	Hum Brain Map	2011	Mutual Information	block	4D	no	30	yes	no	gamma	yes	synthetic	Gaussian	temporal
2	Allen	NeuroImage	2012	ICA	rest	3D	yes	1	yes	yes	canonical	no	synthetic	Rician	none
3	Andrade	Hum Brain Map	2001	Cortical Surface Mapping	rest	2D	no	500	yes	no	square wave	no	synthetic	Gaussian	spatial
4	Backfrieder	Phys Med Bio	1996	PCA	block	3D	no	1	yes	no	square wave	no	synthetic	Gaussian	none
5	Bai	Stat Sin	2008	ICA	block	4D	no	1	no	yes	sinusoidal	no	synthetic	Uniform	none
														+physiological	
6	Bellec	Magn Res Imag	2009	Parametric Bootstrap	block	4D	yes	1	yes	yes	canonical	yes	synthetic	Gaussian + drift	none
														+physiological	
7	Bellec	NeuroImage	2010	cluster analysis	rest	3D	yes	1	yes	no	none	–	synthetic	Gaussian	temporal
8	Birn	NeuroImage	2004	GLM	ER	1D	no	1	yes	yes	gamma	yes	synthetic	Gaussian	none
														+motion	
9	Biswal	J Comp Ass Tom	1999	ICA	rest	3D	no	1	no	yes	sinusoidal	no	synthetic	Gaussian	none
10	Brezger	J Roy Stat Soc C	2007	spatial smoothing	block	3D	no	1	no	no	square wave	no	synthetic	Gaussian	spatial
															& temporal
11	Cabella	Braz J Phys	2008	wavelets	ER	1D	no	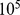	no	no	canonical	no	synthetic	Gaussian	none
12	Cabella	Phys A	2009	wavelets	block	1D	no	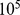	yes	yes	canonical	no	synthetic	Gaussian	none
13	Calhoun	J VLSI	2006	ICA	rest	1D	no	30	yes	no	sinusoidal	no	synthetic	Gaussian	none
14	Calhoun	NeuroImage	2004	GLM	block	1D	no	1	yes	no	canonical	yes	synthetic	Gaussian	none
15	Calhoun	NeuroImage	2005	Spatio-temporal	rest	3D	no	100	yes	no	sinusoidal	no	synthetic	Gaussian	spatial
16	Casanova	NeuroImage	2008	BOLD estimation	ER	1D	no	200	yes	no	canonical	no	synthetic	Gaussian + drift	temporal
17	Casanova	Physio Meas	2009	BOLD estimation	ER	3D	no	200	yes	no	canonical	no	synthetic	Gaussian + drift	none
18	Chen	IEEE T Biomed Eng	2006	ICA	block	3D	no	1	no	yes	square wave	no	synthetic	Gaussian	none
19	Chen	Magn Res Imag	2004	ICA	block	3D	no	1	yes	no	none	–	synthetic	Gaussian	none
20	Chen	Brain Topo	2003	cluster analysis	block	3D	no	1	yes	no	square wave	no	synthetic	Gaussian	none
21	Chen	NeuroImage	2003	t-test	block	4D	no	1	no	yes	square wave	no	hybrid	–	–
22	Churchill	Plos One	2012	preprocessing	block	3D	no	500	yes	no	canonical	no	synthetic	Gaussian	spatial
23	De Martino	NeuroImage	2008	classification	block	4D	no	1	yes	yes	canonical	yes	synthetic	Gaussian	temporal
24	De Mazière	J Magn Res	2007	non-parametric	block	1D	no	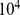	yes	yes	canonical	no	synthetic	Gaussian	temporal
25	Den Dekker	IEEE T Med Imag	2009	LRT	block	1D	no	1000	yes	yes	canonical	no	synthetic	Gaussian	temporal
26	Desco	Hum Brain Map	2001	wavelets	block	3D	no	4	yes	yes	square wave	no	synthetic	Gaussian	spatial
27	Deshpande	IEEE T Biomed Eng	2010	Granger causality	rest	1D	no	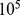	yes	no	canonical	no	synthetic	Gaussian	temporal
28	Desmond	J Neurosc Meth	2002	GLM	block	1D	yes	1000	yes	yes	square wave	no	synthetic	Gaussian	none
29	Dimitriadou	Art Intel Med	2004	cluster analysis	block	3D	no	1	yes	yes	square wave	no	synthetic	Gaussian	none
30	Esposito	Curr Opinion Neuro	2011	ICA	rest	4D	no	2	yes	no	none	–	synthetic	Gaussian	none
31	Fadili	Med Imag Anal	2001	cluster analysis	block	3D	no	1	yes	yes	Poisson	no	synthetic	Gaussian + drift	none
														+physiological	
32	Sun	IEEE T Inf Biomed	2010	GLM	block	4D		1	yes	no	square wave	no	hybrid	–	–
33	Gavrilescu	NeuroImage	2002	Granger causality	rest	1D	no	200	no	no	gamma	yes	synthetic	Gaussian	none
34	Goebel	Magn Res Imag	2003	adaptive thresholding	block	3D	no	500	no	yes	square wave	no	synthetic	Gaussian	spatial
35	Gorgolewski	Front Hum Neurosc	2012	GLM	ER	1D	no	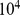	yes	yes	canonical	no	synthetic	Gaussian	temporal
36	Grinband	NeuroImage	2008	Bayesian inference	ER	3D	no	NA	yes	no	canonical	yes	synthetic	Gaussian	none
37	Groves	NeuroImage	2009	ICA	ER	4D	no	1	yes	yes	gamma	yes	hybrid	–	–
38	Gu	NeuroImage	2001	cluster analysis	none	3D	yes	1	no	yes	estimated	no	hybrid	–	–
39	Guo	Stat Int	2010	ICA	rest	3D	yes	1	yes	yes	sinusoidal	no	synthetic	Gaussian	none
40	Guo	NeuroImage	2008	cluster analysis	block	3D	no	100	yes	no	square wave	no	synthetic	Gaussian	spatial
41	Heller	NeuroImage	2006	ICA	block	4D	no	10	yes	no	gamma	yes	hybrid	–	–
42	Hu	NeuroImage	2005	STAP algorithm	block	4D	no	7	yes	no	square wave	no	hybrid	–	–
43	Huang	IEEE T Biomed Eng	2009	cluster analysis	block	3D	no	1	yes	no	gamma	yes	hybrid	–	–
44	Jahanian	Magn Res Imag	2004	connectivity	block	4D	no	15	yes	no	canonical	no	synthetic	Gaussian	none
45	Joel	Magn Res Med	2011	BOLD estimation	ER	1D	no	100	yes	yes	Balloon	no	synthetic	Gaussian	none
46	Johnston	NeuroImage	2008	denoising	ER	3D	no	1	yes	no	square wave	yes	hybrid	–	–
47	Kadah	IEEE T Biomed Eng	2004	mixed effects	block	1D	no	500	no	yes	square wave	no	synthetic	Gaussian	spatial
															& temporal
48	Kang	J Am Stat Ass	2012	connectivity	block	1D		1	yes	no	gamma	yes	synthetic	none	none
49	Kim	Magn Res Imag	2008	GLM	ER	3D	no	1	yes	yes	canonical	no	synthetic	Gaussian	temporal
50	Kim	Int J Imag Sys Tech	2011	fractal scaling	ER	3D	no	1	yes	yes	gamma	no	hybrid	–	–
51	Lee	NeuroImage	2008	GLM	block	3D	no	1	no	no	square wave	no	synthetic	Gaussian	none
52	Lee	IEEE T Med Imag	2011	ICA	block	3D	no	100	yes	yes	square wave	no	synthetic	Uniform + drift	temporal
														+physiological	
53	Lee	J Am Stat Ass	2011	ICA	block	3D		1	no	no	gamma	yes	synthetic	Gaussian	none
54	Lei	NeuroImage	2010	ICA	ER	4D		1	yes	no	canonical	yes	hybrid	–	–
55	LeVan	Hum Brain Map	2009	GLM	ER	3D	yes	400	yes	no	square wave	no	synthetic	Gaussian	spatial
56	Liao	IEEE T Med Imag	2005	GLM	ER	3D	yes	1000	yes	yes	square wave	no	synthetic	Gaussian	spatial
														+Chi-square	
57	Liao	Mag Res Med	2006	ICA	rest	3D	no	1	no	no	sinusoidal	yes	synthetic	super Gaussian	spatial
58	Liao	IEEE T Med Imag	2008	ICA	rest	4D	no	1	yes	yes	sinusoidal	yes	synthetic	super Gaussian	spatial
59	Lindquist	Hum Brain Map	2008	cluster analysis	ER	3D	no	1	yes	no	square wave	yes	synthetic	Gaussian	none
60	Lindquist	NeuroImage	2007	PCA/ICA	block	2D	no	30	no	no	canonical	no	synthetic	mixture Gaussian	temporal
61	Lin	Mag Res Med	2005	t-test	block	4D	no	50	yes	no	square wave	no	hybrid	–	–
62	Lin	NeuroImage	2003	ICA	rest	3D	no	1	yes	yes	canonical	no	synthetic	Gaussian	none
63	Lin	Hum Brain Map	2010	spatial smoothing	block	3D	no	1000	no	no	square wave	no	synthetic	Gaussian	none
64	Li	IEEE T Med Imag	2012	change-point theory	block	3D	no	1	no	no	square wave	no	synthetic	Gaussian	temporal
65	Li	J Roy Stat Soc B	2011	GLM	block	3D	no	1000	yes	yes	square wave	no	synthetic	Gaussian	spatial
66	Logan	NeuroImage	2004	residual analysis	block	1D	no	1000	yes	no	canonical	no	synthetic	Gaussian	none
67	Loh	Stat Sin	2008	ICA	block	3D	no	50	yes	no	canonical	no	synthetic	Gaussian	none
68	Long	Hum Brain Map	2009	GLM	block	1D	no	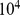	yes	yes	square wave	no	synthetic	Gaussian + drift	none
69	Lowe	J Comp Ass Tom	1999	cluster analysis	ER	3D	no	1	yes	no	gamma	no	synthetic	Gaussian	none
70	Lu	J Mag Res Imag	2006	spatio-temporal	block	3D	no	1	yes	no	canonical	no	synthetic	Gaussian	none
71	Luo	Int J Neural Sys	2006	correlation	block	1D	no	1000	yes	yes	gamma	no	synthetic	Gaussian + drift	none
72	MacIntosh	Hum Brain Map	2003	BOLD estimation	ER	1D	no	1000	yes	no	canonical	no	synthetic	Gaussian + drift	none
73	Marrelec	Hum Brain Map	2003	ICA	rest	3D	yes	1	yes	no	sinusoidal	no	synthetic	super Gaussian	none
74	Moosmann	Int J Psychophysio	2008	spectral analysis	rest	3D	no	1000	yes	yes	sinusoidal	yes	synthetic	Gaussian	temporal
75	Müller	J Mag Res Imag	2007	LRT	block	3D	no	1	no	no	square wave	no	synthetic	Gaussian	none
76	Nan	IEEE T Med Imag	1999	spatio-temporal	block	3D	no	1	no	no	sinusoidal	no	hybrid	–	–
77	Ngan	Mag Res Imag	2001	spatial decomposition	block	4D	yes	500	yes	yes	square wave	no	synthetic	Gaussian	none
78	Park	NeuroImage	2012	t-test	block	1D	no	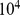	yes	yes	square wave	no	synthetic	Gaussian	none
79	Parrish	Mag Res Med	2000	GLM	block	3D	no	100	yes	no	square wave	no	hybrid	–	–
80	Pendse	NeuroImage	2009	connectivity	ER	1D	no	1000	yes	yes	canonical	no	synthetic	Gaussian	none
81	Penny	NeuroImage	2011	BOLD estimation	ER	3D	no	80	yes	yes	gamma	yes	synthetic	Gaussian + drift	none
82	Puthussery	IEEE T Biomed Eng	2010	spatio-temporal	block	3D	no	1	no	yes	gamma	no	hybrid	–	–
83	Quirós	NeuroImage	2010	spatio-temporal	block	3D	no	1	no	no	gamma	no	hybrid	–	–
84	Quirós	NeuroImage	2010	conditional maximisation	ER	1D	no	100	yes	yes	canonical	no	synthetic	Gaussian	none
85	Rodriguez	NeuroImage	2010	connectivity	ER	1D	no	25	yes	yes	canonical	yes	synthetic	Gaussian	temporal
86	Ryali	NeuroImage	2011	cluster analysis	rest	3D	no	500	no	no	none	–	synthetic	Gaussian	spatial
87	Salli	IEEE T Med Imag	2001	connectivity	block	1D	no	1000	no	no	none	–	synthetic	Gaussian	temporal
88	Sato	NeuroImage	2006	Support Vector Machine	block	3D	yes	100	no	yes	square wave	no	synthetic	Gaussian	none
89	Sato	J Neuro Meth	2008	Granger causality	rest	1D	no	200	yes	yes	canonical	yes	synthetic	Gaussian	temporal
														+physiological	
90	Schippers	NeuroImage	2011	ICA	rest	3D	no	50	no	yes	none	–	synthetic	Gaussian	none
91	Schmithorst	J Mag Res Imag	2009	ICA	rest	4D	yes	1	no	yes	none	–	synthetic	Gaussian	none
92	Schmithorst	J Mag Res Imag	2004	LRT	block	1D	no	NA	no	no	square wave	no	synthetic	Rician	none
93	Sijbers	Med Imag	2004	GLM	block	1D	no	NA	no	no	square wave	no	synthetic	Rician	none
94	Sijbers	Adv Con IVS	2005	LRT	block	1D	no	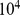	yes	no	square wave	no	synthetic	Gaussian	temporal
95	Sijbers	IEEE T Med Imag	2005	connectivity	block	1D	no	20	yes	yes	canonical	no	synthetic	Gaussian	none
96	Stephan	NeuroImage	2008	GLM	ER	3D	no	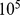	yes	yes	canonical	yes	synthetic	Gaussian	none
97	Sturzbecher	Phys Med Bio	2009	permutation tests	block	3D	no	NA	no	no	square wave	no	synthetic	Gaussian	spatial
															& temporal
98	Suckling	Hum Brain Map	2004	cluster analysis	block	4D	no	1	no	no	canonical	no	synthetic	Gaussian	none
99	Sun	Med Bio Eng Comp	2009	cluster analysis	block	4D	no	1	no	no	canonical	no	synthetic	Gaussian	none
100	Tabelow	IEEE T Med Imag	2008	spatial smoothing	block	3D	no	1	yes	no	canonical	no	synthetic	Gaussian	spatial
															& temporal
101	Thompson	J Mag Res Imag	2006	STAP algorithm	block	3D	no	15	no	no	square wave	no	hybrid	–	–
102	Thompson	J Mag Res Imag	2004	STAP algorithm	block	3D	no	100	no	no	square wave	no	hybrid	–	–
103	Vahdat	Neural Comp	2012	ICA	rest	3D	yes	800	yes	yes	canonical	no	synthetic	Rician	spatial
104	Valdés-Sosa	Phil T Roy Soc Lond B	2005	connectivity	rest	1D	no	NA	no	no	none	–	synthetic	Gaussian	temporal
105	Valente	Mag Res Imag	2009	ICA	block	4D	no	1	yes	yes	canonical	yes	hybrid	–	–
106	Vincent	IEEE T Med Imag	2010	adaptive mixture modelling	ER	3D	no	100	yes	yes	canonical	no	synthetic	Gaussian + drift	none
107	Visscher	NeuroImage	2003	GLM	block	4D	yes	1	no	yes	gamma	no	synthetic	Gaussian	temporal
108	Wager	NeuroImage	2005	robust regression	rest	1D	no	2000	yes	yes	none	–	synthetic	Gaussian	none
109	Wang	NeuroImage	2009	GLM	block	4D	yes	1	yes	yes	canonical	yes	hybrid	–	–
110	Weeda	Hum Brain Map	2009	GLM	block	3D	no	1000	yes	yes	square wave	no	synthetic	Gaussian	none
111	Weeda	NeuroImage	2011	connectivity	rest	4D	no	100	yes	no	none	–	synthetic	Gaussian	none
112	Worsley	NeuroImage	1997	Canonical Variates Analysis	block	4D	no	100	no	no	canonical	yes	synthetic	Gaussian	spatial
															& temporal
113	Xie	Neurocomp	2009	dimension estimation	block	1D	no	20000	yes	no	square wave	no	synthetic	Gaussian	temporal
114	Yue	Stat Int	2010	spatial smoothing	block	3D	no	1	no	no	canonical	no	synthetic	Gaussian	none
115	Zhang	J Multi Anal	2010	classification	ER	1D	no	1000	yes	no	canonical	no	synthetic	Gaussian	temporal
116	Zhang	Ann Stat	2008	BOLD estimation	ER	4D	no	1	yes	yes	estimated	no	synthetic	Gaussian + drift	temporal
117	Zhang	Comp Stat Dat Anal	2008	BOLD estimation	ER	4D	no	500	yes	yes	canonical	no	synthetic	Gaussian + drift	temporal
118	Zhang	IEEE T Biomed Eng	2011	cluster analysis	block	3D	no	1	no	yes	square wave	yes	synthetic	Gaussian	none
119	Zhang	NeuroImage	2012	BOLD estimation	block	1D	yes	100	yes	yes	canonical	yes	synthetic	Gaussian	temporal

ID - paper identification number; Auth. - first author; dim. - data dimension; nS - Multiple subjects?

rep - Number of replications; parV - Parameter variation?; parJ - Parameter justification; HRFm - HRF model; HRFv - HRF variation?; Noise corr. - Noise correlations.

## Results

### Study goals

**Figure 2:**
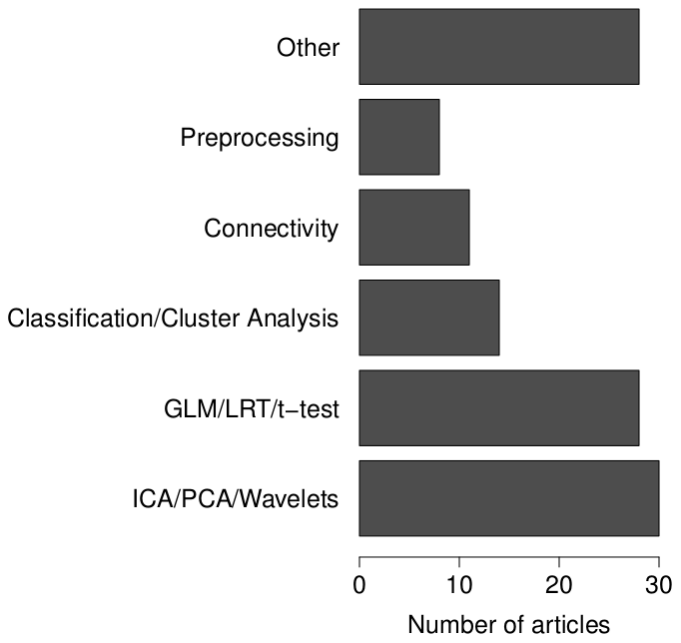
Statistical models investigated in the selected articles.

Simulation studies are conducted to evaluate statistical models based on a given experimental design. For each article we assessed which statistical technique was validated. Six categories of statistical models were distinguished (see Figure 2, left panel). Most simulation studies are conducted for signal decomposition models like Principal Components Analysis (PCA), Independent Component Analysis (ICA) and Wavelet analysis. This group of methods is closely followed by General Linear Model (GLM) analysis, Likelihood Ratio Tests (LRT) and 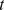-tests. 11.8% of the simulation studies investigate properties of classification techniques using for example Support Vector Machines or cluster analysis. Methods that are less represented in our sample are connectivity analyses or preprocessing methods like motion correction and spatial smoothing. All studies that did not use any of the previous methods were gathered in a “rest” category. In this category are included, for example, HRF estimation methods, spatio-temporal models, bootstrapping and nonparametric techniques.

### Experimental design

The methods described above are validated using a given experimental design (Table 3a). The majority of simulation studies report using a block design for the generation of the BOLD activity. When this design is not used, modelled activation is based on an event-related design or it concerns a resting-state study.

**Table 3:** Proportions of (a) experimental designs, (b) dimensions of the simulated data, and (c) the use of correlated noise reported in the selected articles.

a. Experimental designs			
Block	Event-related	Resting-state	
58.0%	21.8%	20.2%	
b. Data dimensions			
1D	2D	3D	4D
28.6%	1.7%	48.7%	21.0%
c. Noise correlations			
None	Temporal	Spatial	Both
58.0%	24.0%	13.0%	5.0%

### Simulation parameters

The general goal of a simulation study is to research a certain outcome (e.g. power, bias, …) under several conditions (e.g. noise level, HRF variability, …). The most common method to achieve this goal is by conducting a Monte Carlo experiment. The simulation reports in our database were evaluated on the dimensions of the simulated data, the number of replications and parameter variation.

#### Data dimensions

fMRI data have in essence four dimensions (i.e. coordinates in an xyz-space and time). However, the majority of articles in our sample published results for 3D data where time series are simulated for all voxels in a single slice (Table 3b), while one fifth considered full 4D fMRI data. On the other hand, many of the articles reported simulating fMRI time series only with no spatial context. In this case, mass-univariate techniques were mostly evaluated that also regard fMRI data as being multiple measurements of single time series. A very small proportion considered two-dimensional data. This was reported exclusively in an ICA validation context, where the fMRI data are organised as *voxels* 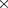 *timepoints*.

#### Replications

**Figure 3:**
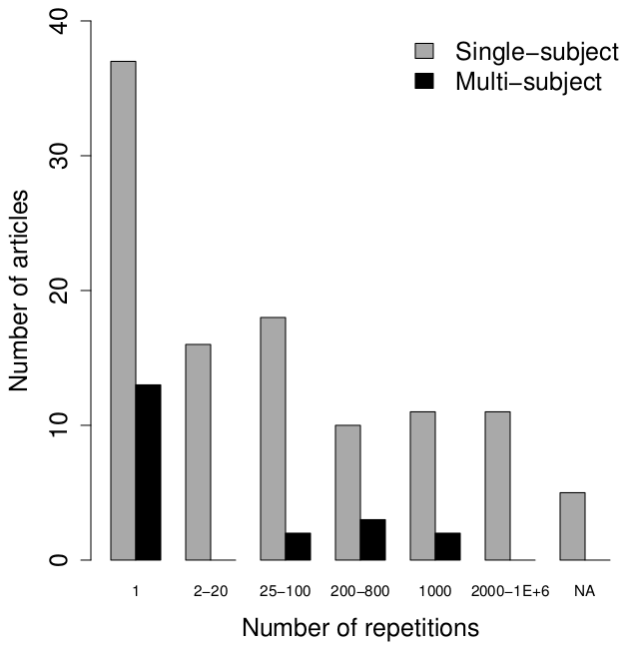
Overview of the number of replications for single-subject and multi-subject simulations.

The overall majority of the selected articles considered single-subject data, while the remaining articles simulated data for multiple subjects. In these last studies, the number of subjects that was simulated corresponded typically with sample sizes reported in real fMRI studies (e.g. 4 to 20 subjects) and the data for these subjects were mostly simulated once (with a few notable exceptions, see Figure 3, right panel). For the single-subject simulation studies, the number of repetitions was higher in the majority of the studies, while about one third of the articles reported 1 replication of the simulated data for each setting of the manipulated parameters. It should be noted that simulating 3D or 4D datasets without any spatial correlations is equal to the simulation of fMRI time series with 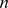 replications where 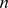 is the number of voxels. This was true for 22 of the 37 studies that reported using 1 replication. However, for the remaining studies conclusions are based on 1 realisation of the data. Two studies reported simulating time series just once for each setting of the simulation parameters.

#### Parameter variation

Other possible parameters taken into account in the simulations were, for example, strength of the modelled activation, number of time points, noise level, repetition time (TR), etc. The relevance of a simulation study is highly dependant on the representativeness of these chosen parameter values. To ensure that the parameters are characteristic for fMRI data, it is recommended that a range of values is evaluated. Additionally, a justification is expected on why specific values of certain parameters are chosen. In our sample both requirements were assessed (see Table 4 for an overview). A study was classified as using varying parameters as soon as more than one value of a specific parameter was considered. Whenever a reason for choosing a specific parameter value was reported, the simulation study was evaluated as positive on the justification of the chosen parameters. About one third of the studies reported a variation in the values and gave a justification for their choices. Frequently reported variations were several noise levels and activation strengths that were taken into account. As for the choices of the values, authors mostly justified these as being realistic values in real fMRI data or being estimated from real data. However, about one third of the studies reporting variation of the parameters did not give any justification, ten percent did justify the choice of the parameter values but only used one specific value for each parameter, while one fifth of the studies in our sample did neither.

**Table 4:** Proportions of studies reporting (a) parameter variation and justification of the chosen parameter values and (b) whether HRF variability was taken into account.

a. Parameter variation and justification		
	Justification of value	
Parameter variation	No	Yes
No	20.2%	10.9%
Yes	32.8%	36.1%
b. HRF variation		
	No	Yes
	77.3%	22.7%

### Data generation models

Of all simulation studies investigated, 84% were pure synthetic simulations while the other 16% adopted a hybrid simulation strategy. In hybrid simulations, a resting-state dataset is acquired and synthetically generated activation is added to these data. As such, knowledge of the ground truth is assured while the noise is representative for real data. However, manipulation of the noise in the simulated fMRI data is not possible and replicating the data will be a costly process. Therefore, in most simulations the fMRI data are generated completely artificially.

All synthetic simulation studies adopted an additive data generation model (e.g. [8]) in which three main components can be distinguished: (1) a baseline signal, (2) BOLD activation and (3) noise. However, half of the studies did not report using a baseline for the data, so we could assume that this is zero for these studies. For the other half, 47% used a static baseline, for example a constant when simulating time series and a template slice or volume that was repeated for each time point in the case of simulating 3D or 4D fMRI data. A minority of the studies (3%) used a varying baseline, meaning that the baseline values were varied over time, e.g. to model thermal shifts [9].

#### BOLD response

**Figure 4:**
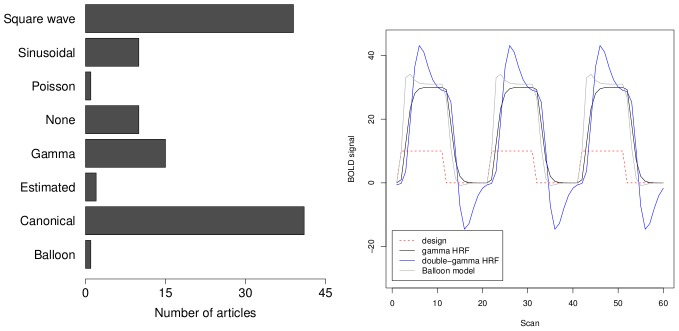
Overview of the different HRF functions used in the simulation studies (left) and illustration of the BOLD response shapes as the result of a block design fMRI experiment for the different HRF models (right, source: [24]).

An important component in the simulated fMRI data is the BOLD response because this signal defines the ground truth in the simulation studies. Despite the fact that the coupling between the neural activation and the BOLD response is still not completely understood [6], several models are available to generate a haemodynamic response function (HRF). See Figure 4 (left) for an overview of the models used in the selected articles. Those methods are, for example, a gamma function [10], [11], a difference of two gamma functions, also known as the canonical HRF [12], [13] or the Balloon model [14], [15]. The different shapes of these models are illustrated in Figure 4 (right). Nevertheless, one third of the reported simulation studies disregarded any BOLD characteristics and chose a square wave (i.e. a boxcar function) to represent the BOLD activation in the simulated fMRI data. When no experimental task was simulated, resting-state activation was predominantly modelled as a set of sinusoidal functions, although a few of the selected studies did not simulate any BOLD activation. The shape of the HRF varies immensely from brain region to brain region and also from subject to subject. One fifth of the simulation studies reported modelling this variation in the HRF parameters, while the majority considered a fixed HRF in all simulations (Table 4b).

#### Noise model

**Figure 5:**
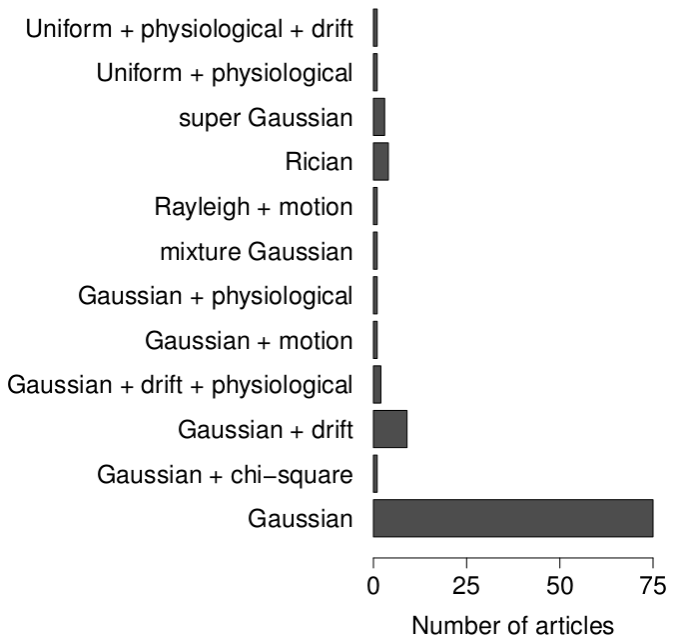
Overview of the noise models in the synthetic simulation studies.

Noise is not only characteristic for fMRI data but also ensures generalisability of the conclusions based on simulations. All simulation studies incorporated some noise generating process (see Figure 5, left panel, for an overview). The vast majority of the synthetic simulation studies (i.e. 75%) selected the noise randomly from a Gaussian distribution. An additional 9% added also some drift function to this noise, while about 7% of the studies considered a skewed noise distribution (e.g. Rician or super Gaussian distribution). The remainder of the studies used a very specific noise model (for example by adding physiological noise, using a uniform distribution or adding motion correlated noise), because they focused on the effects of these noise sources. fMRI noise is also known to be spatially and temporally correlated. However, the majority of the selected articles did not report modelling any correlations in the noise (Table 3c). Temporal correlation was almost exclusively modelled as an auto-regressive autocorrelation process. Typically this process was of order 1, but there are exceptions that used a model order of 3 or 4. Spatial correlations were typically created by spatial smoothing of the generated noise. A small fraction of the studies modeled both spatial and temporal correlations.

## Discussion

Whenever statistical models are validated based on simulations, the model that is used for the data generation is of utmost importance. In this paper, a survey was conducted to list currently used data generation models. Based on 119 research articles we described the simulation type, use and justification of simulation parameters and the different components in the fMRI data generating process. The survey results showed that current fMRI simulation studies sometimes lack a thorough experimental manipulation. The parameters in the simulation study (e.g. noise level, TR, HRF delay, etc.) are not always varied, while representative values of some of these parameters are not known. Using the results from iEEG could guide many of these parameter choices and make simulation more realistic in general. Further, the number of replications is a major topic of concern. We observed that the conclusions of some of the simulation studies were based on only one replication of the random data generating process. When a simulation study is used to evaluate the expectation of random variables, the external validity of the study is threatened if only a few replications of the data generating process are used.

### Model-based versus data-based simulation

In about 60% of the synthetic simulation studies, the fMRI data were generated based on a model similar to the model being validated (e.g. generating time series from a VAR model to evaluate Granger causality). As such, the simulation is entirely model-based and the assumptions of the model under investigation are completely met. Consequently, the conclusions of these simulation studies give only partial information on the applicability of these models as an analysis tool for fMRI data, since fMRI data generally do not meet the assumptions of most statistical models. A better practice would be to start from the data themselves and to define a data generating process that models the different sources that are present in fMRI data. By using data-based simulations, the properties of the analysis techniques can be assessed in more realistic circumstances.

In this context, it should be noted that the data generating process used in most current simulation studies is not compatible with the knowledge on how fMRI data are constructed. For instance, it is well-known that the BOLD response is the result of a haemodynamic coupling to neural activity. Although the precise dynamics are perhaps still debatable, there is consensus about the BOLD signal being a delayed response with varying dynamics over the brain regions and between subjects. However, about one third of the reported simulation studies in our database did not model any of these characteristics and used a simple boxcar function to distinguish stimulus induced activation from rest (Figure 4). About the same number did model the slow emergence of the BOLD signal by using a canonical HRF, but only a small fraction (i.e. two studies) did also model BOLD nonlinearities by means of the Balloon model. In the case of spontaneous neural activation (for example in resting-state studies), BOLD fluctuations were mostly modelled through sinusoidal functions with frequencies that are commonly observed in resting-state studies. However, describing these spontaneous fluctuations by sinusoids stems from the tradition to use ICA to analyse these data and is again more compatible with the model under investigation than being representative for the data. Further, variability of the BOLD response was taken into account only in about one fifth of the simulation studies (Table 4b). With regard to modelling BOLD activation, in a data-based simulation context at least some form of HRF should be used that takes into account the basic characteristics of the BOLD signal, while any variation of the parameters of this model will enhance the generalisability of the simulation results.

The generation of fMRI noise alsocauses a discrepancy between simulated and real fMRI data. The noise in fMRI consists of several sources [7], [16], for example thermal noise, motion related noise, physiological noise and task-related noise. Nevertheless, the vast majority of simulation studies investigated here have only used a white Gaussian noise model to generate fMRI noise ignoring its multiple-source character. In some cases, spatial or temporal correlations are added. Again, this noise model is consistent with many of the statistical models for fMRI data (e.g. GLM). Unfortunately, the Gaussian noise model only accounts for a fraction of the noise in real data. One solution is to use hybrid simulations in which using real noise acquired in a resting-state study increases the realistic character of the simulated data. However, it is impossible to manipulate noise related parameters and unwanted activation in resting-state data can influence the simulation results. Moreover, multiple replications (i.e. acquiring resting-state data from multiple subjects) are costly. Perhaps the better solution is to model more than only Gaussian noise (i.e. thermal noise) and also include, as has been demonstrated in several simulation studies, motion noise, physiological noise, signal drift, etc. In some simulation studies, the results will not be altered under a full noise model. It may not always be necessary to include all noise sources (e.g. if a certain noise source is removed or the influence of a source is assumed to be equal in all conditions), but this should be motivated at least. To assure generalisability of the simulation results, a more complex noise model, compared to the one that is generally adopted now, might be imperative.

### Recommendations for simulating fMRI data

Based on these results we present some recommendations to improve the reliability and generalisability of fMRI simulation studies.

1. All parameters for which a value is chosen in the simulation experiments should be thoroughly justified. If a single value is not agreed upon, a range of values should be evaluated (see [8], [17]–[19] for some examples).
2. The conditions in the simulation study, (e.g. statistical model, parameter values,…), have to be combined in an experimental design. The construction of this experimental design is essential [20]. Factors that can be considered in the experiment are, for example, variations of parameter levels, analysis methods and number of replications. The most complete design is the full-factorial design, although there might be reasons to adopt fractional designs. Based on the experimental design, the simulation experiment will have external validity (i.e. the results can be generalised beyond a given experiment).
3. A Monte Carlo experiment has to be repeated to exclude random influences on the simulation results. Therefore, a sufficient number of replications of the experiment has to be performed. In the case of time series simulations, at least 10000 replications might be necessary, while for the simulation of 3D or 4D fMRI data a total of 100 might be enough. In general, the more replications, the better. For example, [19] generated 10000 replications of 3D datasets, and [17] simulated 4D multi-subject datasets to represent twin data using 500 replications of each paired dataset. In practice, this number can be limited due to time or computational constraints. When in doubt, the convergence of the results should be tested.
4. The simulated task-related activation signal should reflect known properties of the BOLD response. This includes, but is not limited to, response delay, nonlinearities and inter-region and -subject variability. Either the canonical HRF or the Balloon model can be used (see [21] for an example using the Balloon model).
5. fMRI noise is partially white (i.e. system noise) and this part can be modelled by random Gaussian noise. However, in addition one should account for (residual) motions, heart rate and respiratory rate fluctuations, task-related noise and spatial and temporal correlations (see, for example, [8], [22], [23]).
6. If either the BOLD model or the noise model is simplified, this should be duly motivated.

### Conclusion

The use of simulation studies to validate statistical techniques for fMRI data should be highly encouraged, because simulation experiments are a fast and cheap tool to assess the quality and applicability of the analysis techniques. However, our survey of the fMRI simulation literature raised several concerns with respect to simulation studies as they are conducted now. The observed decrease in the number of fMRI simulation studies in recent years is troubling. Furthermore, it was demonstrated that the data generating process used to simulate fMRI data is often model-based and parameter variation in the data generating process is not implemented in a standard manner.

A possible reason for the absence of a common fMRI data generation model might be the lack of established software packages. Current simulation studies are mainly conducted using in-house software routines that have no common programming language and are not widely available. Recently, developments to fill this gap have resulted in the release of software packages that provide a flexible and fast framework for fMRI simulations [24], [25]. Using these software packages can be an important step in the right direction. Additionally, by taking into account the different sources present in fMRI data and adopting a complete simulation design with sufficient replications, conclusions from fMRI simulation studies can be expected to be more reliable.

Researchers that conduct fMRI simulation studies are encouraged to consider the recommendations presented in this paper in order to increase the reliability and generalisability of the conclusions from simulation studies.

## Supporting Information

Table S1 **Bibliographic details of the selected articles.**
(PDF)
