# Supplementary material for: A Review of fMRI Simulation Studies
Source: PLoS One. 2014 Jul 21;9(7):e101953. doi: 10.1371/journal.pone.0101953 (PMC4105464; doi:10.1371/journal.pone.0101953)
Supplement: Table S1 — Bibliographic details of the selected articles. (PDF) [file pone.0101953.s001.pdf]

[1] Afshin-Pour B, Soltanian-Zadeh H, Hossein-Zadeh GA, Grady C, Strother S (2011) A mutual information-based metric for evaluation of fMRI data-processing approaches. Human Brain Mapping 32: 699–715.

[2] Allen E, Erhardt E, Wei Y, Eichele T, Calhoun V (2012) Capturing inter-subject variability with group independent component analysis of fMRI data: A simulation study. NeuroImage 59: 4141–59.

[3] Andrade A, Kherif F, Mangin J, Worsley K, Paradis A, et al. (2001) Detection of fMRI activation using cortical surface mapping. Human Brain Mapping 12: 79–93.

[4] Backfrieder W, Baumgartner R, Sámal M, Moser E, Bergmann H (1996) Quantification of intensity variations in functional MR images using rotated principal components. Physics in Medicine and Biology 41: 1425–1438.

[5] Bai P, Shen H, Huang X, Truong Y (2008) A supervised singular value decomposition for independent component analysis of fMRI. Statistica Sinica 18: 1233–1252.

[6] Bellec P, Perlbarg V, Evans A (2009) Bootstrap generation and evaluation of an fMRI simulation database. Magnetic Resonance Imaging 27: 1382–1396.

[7] Bellec P, Rosa-Neto P, Lyttelton O, Benali H, Evans A (2010) Multilevel bootstrap analysis of stable clusters in resting-state fMRI. NeuroImage 51: 1126–1139.

[8] Birn R, Cox R, Bandettini P (2004) Experimental designs and processing strategies for fMRI studies involving overt verbal responses. NeuroImage 23: 1046–1058.

[9] Biswal B, Ulmer J (1999) Blind source separation of multiple signal sources of fMRI data sets using independent component analysis. Journal Of Computer Assisted Tomography 23: 265–271.

[10] Brezger A, Fahrmeir L, Hennerfeind A (2007) Adaptive Gaussian Markov random fields with applications in human brain mapping. Journal of the Royal Statistical Society: Series C (Applied Statistics) 56: 327—-345.

[11] Cabella B, Sturzbecher M, Tedeschi W, Baffa Filho O, de Araa D, et al. (2008) A numerical study of the Kullback-Leibler distance in functional magnetic resonance imaging. Brazilian Journal of Physics 38: 20 – 25.

[12] Cabella B, Sturzbecher M, De Araujo D, Neves U (2009) Generalized relative entropy in functional magnetic resonance imaging. Physica A: Statistical Mechanics and its Applications 388: 41–50.

[13] Calhoun V, Adali T (2006) Complex infomax: Convergence and approximation of Infomax with complex nonlinearities. Journal of VLSI Signal Processing Systems for Signal Image and Video Technology 44: 173-190.

[14] Calhoun V, Stevens M, Pearlson G, Kiehl K (2004) fMRI analysis with the general linear model: removal of latency-induced amplitude bias by incorporation of hemodynamic derivative terms. NeuroImage 22: 252–257.

[15] Calhoun V, Adali T, Stevens M, Kiehl K, Pekar J (2005) Semi-blind ICA of fMRI: A method for utilizing hypothesis-derived time courses in a spatial ICA analysis. NeuroImage 25: 527–538.

[16] Casanova R, Ryali S, Serences J, Yang L, Kraft R, et al. (2008) The impact of temporal regularization on estimates of the BOLD hemodynamic response function: a comparative analysis. NeuroImage 40: 1606–1618.

[17] Casanova R, Yang L, Hairston W, Laurienti P, Maldjian J (2009) Evaluating the impact of spatio-temporal smoothness constraints on the BOLD hemodynamic response function estimation: an analysis based on Tikhonov regularization. Physiological Measurement 30: N37–N51.

[18] Chen H, Yuan H, Yao D, Chen L, Chen W (2006) An integrated neighborhood correlation and hierarchical clustering approach of functional MRI. IEEE Transactions on Biomedical Engineering 53: 452–458.

[19] Chen H, Yao D (2004) Discussion on the choice of separated components in fMRI data analysis by spatial independent component analysis. Magnetic Resonance Imaging 22: 827–833.

[20] Chen H, Yao D, Zhuo Y, Chen L (2003) Analysis of fMRI data by blind separation of data in a tiny spatial domain into independent temporal component. Brain Topography 15: 223–232.

[21] Chen NK, Dickey C, Yoo SS, Guttmann C, Panych L (2003) Selection of voxel size and slice orientation for fMRI in the presence of susceptibility field gradients: application to imaging of the amygdala. NeuroImage 19: 817–825.

[22] Churchill N, Yourganov G, Oder A, Tam F, Graham S, et al. (2012) Optimizing Preprocessing and Analysis Pipelines for Single-Subject fMRI: 2. Interactions with ICA, PCA, Task Contrast and Inter-Subject Heterogeneity. PLoS ONE 7: e31147.

[23] De Martino F, Valente G, Staeren N, Ashburner J, Goebel R, et al. (2008) Combining multivariate voxel selection and support vector machines for mapping and classification of fMRI spatial patterns. NeuroImage 43: 44–58.

[24] De Mazière P, Van Hulle M (2007) fMRI bold signal analysis using a novel nonparametric statistical method. Journal of Magnetic Resonance 185: 138–151.

[25] Den Dekker A, Poot D, Bos R, Sijbers J (2009) Likelihood-based hypothesis tests for brain activation detection from MRI data disturbed by colored noise: a simulation study. IEEE Transactions on Medical Imaging 28: 287–296.

[26] Desco M, Hernandez J, Santos A, Brammer M (2001) Multiresolution analysis in fMRI: sensitivity and specificity in the detection of brain activation. Human Brain Mapping 14: 16–27.

[27] Deshpande G, Sathian K, Hu X (2010) Assessing and Compensating for Zero-Lag Correlation Effects in Time-Lagged Granger Causality Analysis of fMRI. IEEE Transactions on Biomedical Engineering 57: 1446–1456.

[28] Desmond J, Glover G (2002) Estimating sample size in functional MRI (fMRI) neuroimaging studies: statistical power analyses. Journal of Neuroscience Methods 118: 115–128.

[29] Dimitriadou E, Barth M, Windischberger C, Hornik K, Moser E (2004) A quantitative comparison of functional MRI cluster analysis. Artificial Intelligence in Medicine 31: 57–71.

[30] Esposito F, Goebel R (2011) Extracting functional networks with spatial independent component analysis: the role of dimensionality, reliability and aggregation scheme. Current Opinion in Neurology 24: 378–385.

[31] Fadili M, Ruan S, Bloyet D, Mazoyer B (2001) On the number of clusters and the fuzziness index for unsupervised FCA application to BOLD fMRI time series. Medical Image Analysis 5: 55–67.

[32] Sun F, Morris D, Lee W, Taylor M, Mills T, et al. (2010) Feature-Space-Based fMRI Analysis Using the Optimal Linear Transformation. Information Technology in Biomedicine, IEEE Transactions on 14: 1279 –1290.

[33] Gavrilescu M, Shaw M, Stuart G, Eckersley P, Svalbe I, et al. (2002) Simulation of the effects of global normalization procedures in functional MRI. NeuroImage 17: 532–542.

[34] Goebel R, Roebroeck A, Kim DS, Formisano E (2003) Investigating directed cortical interactions in time-resolved fMRI data using vector autoregressive modeling and Granger causality mapping. Magnetic Resonance Imaging 21: 1251–1261.

[35] Gorgolewski K, Storkey A, Bastin M, Pernet C (2012) Adaptive thresholding for reliable topological inference in single subject fMRI analysis. Frontiers in Human Neuroscience 6.

[36] Grinband J, Wager T, Lindquist M, Ferrera V, Hirsch J (2008) Detection of time-varying signals in event-related fMRI designs. NeuroImage 43: 509–520.

[37] Groves A, Chappell M, Woolrich M (2009) Combined spatial and non-spatial prior for inference on MRI time-series. NeuroImage 45: 795–809.

[38] Gu H, Engelien W, Feng H, Silbersweig D, Stern E, et al. (2001) Mapping transient, randomly occurring neuropsychological events using independent component analysis. NeuroImage 14: 1432–1443.

[39] Guo Y (2010) A weighted cluster kernel PCA prediction model for multi-subject brain imaging data. Statistics and Its Interface 3: 103–111.

[40] Guo Y, DuBois Bowman F (2008) Modeling dose-dependent neural processing responses using mixed effects spline models: with application to a PET study of ethanol. NeuroImage 40: 698–711.

[41] Heller R, Stanley D, Yekutieli D, Rubin N, Benjamini Y (2006) Cluster-based analysis of FMRI data. NeuroImage 33: 599–608.

[42] Hu D, Yan L, Liu Y, Zhou Z, Friston K, et al. (2005) Unified SPM-ICA for fMRI analysis. NeuroImage 25: 746–755.

[43] Huang L, Thompson E, Schmithorst V, Holland S, Talavage T (2009) Partially adaptive STAP algorithm approaches to functional MRI. IEEE Transactions on Biomedical Engineering 56: 518–521.

[44] Jahanian H, Hossein-Zadeh GA, Soltanian-Zadeh H, Ardekani B (2004) Controlling the false positive rate in fuzzy clustering using randomization: application to fMRI activation detection. Magnetic Resonance Imaging 22: 631–638.

[45] Joel S, Caffo B, Van Zijl P, Pekar J (2011) On the relationship between seed-based and ICA-based measures of functional connectivity. Magnetic Resonance in Medicine 66: 644–657.

[46] Johnston L, Duff E, Mareels I, Egan G (2008) Nonlinear estimation of the BOLD signal. NeuroImage 40: 504–514.

[47] Kadah Y (2004) Adaptive denoising of event-related functional magnetic resonance imaging data using spectral subtraction. IEEE Transactions on Biomedical Engineering 51: 1944–1953.

[48] Kang H, Ombao H, Linkletter C, Long N, Badre D (2012) Spatio-Spectral Mixed-Effects Model for Functional Magnetic Resonance Imaging Data. Journal of the American Statistical Association 107: 568–577.

[49] Kim B, Yeo D, Bhagalia R (2008) Comprehensive mathematical simulation of functional magnetic resonance imaging time series including motion-related image distortion and spin saturation effect. Magnetic Resonance Imaging 26: 147–159.

[50] Kim E, Han Y, Park H (2011) New fMRI analysis method for multiple stimuli using reference estimation. International Journal of Imaging Systems and Technology 21: 315—-322.

[51] Lee JM, Hu J, Gao J, Crosson B, Peck K, et al. (2008) Discriminating brain activity from task-related artifacts in functional MRI: fractal scaling analysis simulation and application. NeuroImage 40: 197–212.

[52] Lee K, Tak S, Ye J (2011) A Data-Driven Sparse GLM for fMRI Analysis Using Sparse Dictionary Learning With MDL Criterion. IEEE Transactions on Medical Imaging 30: 1076–1089.

[53] Lee S, Shen H, Truong Y, Lewis M, Huang X (2011) Independent Component Analysis Involving Autocorrelated Sources With an Application to Functional Magnetic Resonance Imaging. Journal of the American Statistical Association 106: 1009–1024.

[54] Lei X, Qiu C, Xu P, Yao D (2010) A parallel framework for simultaneous EEG/fMRI analysis: methodology and simulation. NeuroImage 52: 1123–1134.

[55] LeVan P, Gotman J (2009) Independent component analysis as a model-free approach for the detection of BOLD changes related to epileptic spikes: a simulation study. Human Brain Mapping 30: 2021–31.

[56] Liao R, Krolik J, McKeown M (2005) An information-theoretic criterion for intrasubject alignment of FMRI time series: motion corrected independent component analysis. IEEE Transactions on Medical Imaging 24: 29–44.

[57] Liao R, McKeown M, Krolik J (2006) Isolation and minimization of head motion-induced signal variations in fMRI data using independent component analysis. Magnetic Resonance in Medicine 55: 1396–1413.

[58] Liao W, Chen H, Yang Q, Lei X (2008) Analysis of fMRI Data Using Improved Self-Organizing Mapping and Spatio-Temporal Metric Hierarchical Clustering. IEEE Transactions on Medical Imaging 27: 1472–1483.

[59] Lindquist M, Wager T (2008) Spatial smoothing in fMRI using prolate spheroidal wave functions. Human Brain Mapping 29: 1276–1287.

[60] Lindquist M, Waugh C, Wager T (2007) Modeling state-related fMRI activity using change-point theory. NeuroImage 35: 1125–1141.

[61] Lin FH, Huang TY, Chen NK, Wang FN, Stufflebeam S, et al. (2005) Functional MRI using regularized parallel imaging acquisition. Magnetic Resonance in Medicine 54: 343–353.

[62] Lin FH, McIntosh A, Agnew J, Eden G, Zeffiro T, et al. (2003) Multivariate analysis of neuronal interactions in the generalized partial least squares framework: simulations and empirical studies. NeuroImage 20: 625–642.

[63] Lin QH, Liu J, Zheng YR, Liang H, Calhoun V (2010) Semiblind spatial ICA of fMRI using spatial constraints. Human Brain Mapping 31: 1076–1088.

[64] Li Y, Gilmore J, Wang J, Styner M, Lin W, et al. (2012) TwinMARM: two-stage multiscale adaptive regression methods for twin neuroimaging data. IEEE Transactions on Medical Imaging 31: 1100–12.

[65] Li Y, Zhu H, Shen D, Lin W, Gilmore J, et al. (2011) Multiscale Adaptive Regression Models for Neuroimaging Data. Journal of the Royal Statistical Society Series B Statistical methodology 73: 559–578.

[66] Logan B, Rowe D (2004) An evaluation of thresholding techniques in fMRI analysis. NeuroImage 22: 95–108.

[67] Loh J, Lindquist M, Wager T (2008) Residual analysis for detecting mis-modeling in fMRI. Statistica Sinica 18: 1421–1448.

[68] Long Z, Chen K, Wu X, Reiman E, Peng D, et al. (2009) Improved application of independent component analysis to functional magnetic resonance imaging study via linear projection techniques. Human Brain Mapping 30: 417–431.

[69] Lowe M, Russell D (1999) Treatment of baseline drifts in fMRI time series analysis. Journal Of Computer Assisted Tomography 23: 463–473.

[70] Lu N, Shan BC, Li K, Yan B, Wang W, et al. (2006) Improved temporal clustering analysis method for detecting multiple response peaks in fMRI. Journal of Magnetic Resonance Imaging 23: 285–290.

[71] Luo S Hand Puthusserypady (2006) Spatio-temporal modeling and analysis of fMRI data using NARX neural network. International Journal of Neural Systems 16: 139–149.

[72] MacIntosh B, Baker S, Mraz R, Ives J, Martel A, et al. (2007) Improving functional magnetic resonance imaging motor studies through simultaneous electromyography recordings. Human Brain Mapping 28: 835–845.

[73] Marrelec G, Benali H, Ciuciu P, Pélégrini-Issac M, Poline JB (2003) Robust Bayesian estimation of the hemodynamic response function in event-related BOLD fMRI using basic physiological information. Human Brain Mapping 19: 1–17.

[74] Moosmann M, Eichele T, Nordby H, Hugdahl K, Calhoun V (2008) Joint independent component analysis for simultaneous EEG-fMRI: principle and simulation. International Journal of Psychophysiology 67: 212–221.

[75] Müller K, Neumann J, Grigutsch M, Von Cramon D, Lohmann G (2007) Detecting groups of coherent voxels in functional MRI data using spectral analysis and replicator dynamics. Journal of Magnetic Resonance Imaging 26: 1642–1650.

[76] Nan F, Nowak R (1999) Generalized likelihood ratio detection for fMRI using complex data. IEEE Transactions on Medical Imaging 18: 320–329.

[77] Ngan S, Auffermann W, Sarkar S, Hu X (2001) Activation detection in event-related fMRI data based on spatio-temporal properties. Magnetic Resonance Imaging 19: 1149–1158.

[78] Park J, Shedden K, Polk T (2012) Correlation and heritability in neuroimaging datasets: a spatial decomposition approach with application to an fMRI study of twins. NeuroImage 59: 1132–42.

[79] Parrish T, Gitelman D, LaBar K, Mesulam M (2000) Impact of signal-to-noise on functional MRI. Magnetic Resonance in Medicine 44: 925–932.

[80] Pendse G, Borsook D, Becerra L (2009) Enhanced false discovery rate using Gaussian mixture models for thresholding fMRI statistical maps. NeuroImage 47: 231–261.

[81] Penny W (2011) Comparing Dynamic Causal Models using AIC, BIC and Free Energy. NeuroImage 59: 319–330.

[82] Puthusserypady S, Ratnarajah T, Jue R (2010) Robust Estimation of HDR in fMRI using H-Filters. IEEE Transactions on Biomedical Engineering 57: 1133–1142.

[83] Quirós A, Diez R, Gamerman D (2010) Bayesian spatiotemporal model of fMRI data. NeuroImage 49: 442–456.

[84] Quirós A, Diez R, Wilson S (2010) Bayesian spatiotemporal model of fMRI data using transfer functions. NeuroImage 52: 995–1004.

[85] Rodriguez P (2010) Using conditional maximization to determine hyperparameters in model-based fMRI. NeuroImage 50: 472–478.

[86] Ryali S, Supekar K, Chen T, Menon V (2011) Multivariate dynamical systems models for estimating causal interactions in fMRI. NeuroImage 54: 807–823.

[87] Salli E, Aronen H, Savolainen S, Korvenoja A, Visa A (2001) Contextual clustering for analysis of functional MRI data. IEEE Transactions on Medical Imaging 20: 403–414.

[88] Sato J, Junior E, Takahashi D, De Maria Felix M, Brammer M, et al. (2006) A method to produce evolving functional connectivity maps during the course of an fMRI experiment using wavelet-based time-varying Granger causality. NeuroImage 31: 187–196.

[89] Sato J, Mourão Miranda J, Morais Martin M, Amaro E, Morettin P, et al. (2008) The impact of functional connectivity changes on support vector machines mapping of fMRI data. Journal of Neuroscience Methods 172: 94–104.

[90] Schippers M, Renken R, Keysers C (2011) The effect of intra- and intersubject variability of hemodynamic responses on group level Granger causality analyses. NeuroImage 57: 22–36.

[91] Schmithorst V (2009) Higher-order contrast functions improve performance of independent component analysis of fMRI data. Journal of Magnetic Resonance Imaging 29: 242–249.

[92] Schmithorst V, Holland S (2004) Comparison of three methods for generating group statistical inferences from independent component analysis of functional magnetic resonance imaging data. Journal of Magnetic Resonance Imaging 19: 365–368.

[93] Sijbers J, den Dekker A (2004) Generalized likelihood ratio tests for complex fMRI data. Medical Imaging 2004: Physiology, Function, and Structure from Medical Images 5: 652-663.

[94] Sijbers J, den Dekker A, Bos R (2005) A likelihood ratio test for functional MRI data analysis to account for colored noise. Advanced Concepts for Intelligent Vision Systems 3708: 538-546.

[95] Sijbers J, Den Dekker AJ (2005) Generalized likelihood ratio tests for complex fMRI data: a Simulation study. IEEE Transactions on Medical Imaging 24: 604–611.

[96] Stephan K, Kasper L, Harrison L, Daunizeau J, Den Ouden H, et al. (2008) Nonlinear dynamic causal models for fMRI. NeuroImage 42: 649–662.

[97] Sturzbecher M, Tedeschi W, Cabella B, Baffa O, Neves U, et al. (2009) Non-extensive entropy and the extraction of BOLD spatial information in event-related functional MRI. Physics in Medicine and Biology 54: 161–174.

[98] Suckling J, Bullmore E (2004) Permutation tests for factorially designed neuroimaging experiments. Human Brain Mapping 22: 193–205.

[99] Sun F, Morris D, Babyn P (2009) The optimal linear transformation-based fMRI feature space analysis. Medical & Biological Engineering & Computing 47: 1119–1129.

[100] Tabelow K, Polzehl J, Ulug A, Dyke J, Watts R, et al. (2008) Accurate Localization of Brain Activity in Presurgical fMRI by Structure Adaptive Smoothing. IEEE Transactions on Medical Imaging 27: 531–537.

[101] Thompson E (2006) A parallel approach to STAP implementation for fMRI data. Journal of Magnetic Resonance Imaging 23: 216–221.

[102] Thompson E, Holland S, Schmithorst V (2004) A STAP algorithm approach to fMRI: a simulation study. Journal of Magnetic Resonance Imaging 20: 715–722.

[103] Vahdat S, Maneshi M, Grova C, Gotman J, Milner T (2012) Shared and Specific Independent Components Analysis for Between-Group Comparison. Neural Computation 24: 3052–3090.

[104] Valdés-Sosa P, Sánchez-Bornot J, Lage-Castellanos A, Vega-Hernández M, Bosch-Bayard J, et al. (2005) Estimating brain functional connectivity with sparse multivariate autoregression. Philosophical Transactions of the Royal Society of London - Series B: Biological Sciences 360: 969–981.

[105] Valente G, De Martino F, Filosa G, Balsi M, Formisano E (2009) Optimizing ICA in fMRI using information on spatial regularities of the sources. Magnetic Resonance Imaging 27: 1110–1119.

[106] Vincent T, Risser L, Ciuciu P (2010) Spatially adaptive mixture modeling for analysis of FMRI time series. IEEE Transactions on Medical Imaging 29: 1059–1074.

[107] Visscher K, Miezin F, Kelly J, Buckner R, Donaldson D, et al. (2003) Mixed blocked/event-related designs separate transient and sustained activity in fMRI. NeuroImage 19: 1694–1708.

[108] Wager T, Keller M, Lacey S, Jonides J (2005) Increased sensitivity in neuroimaging analyses using robust regression. NeuroImage 26: 99–113.

[109] Wang Z (2009) A hybrid SVM-GLM approach for fMRI data analysis. NeuroImage 46: 608–615.

[110] Weeda W, Waldorp L, Christoffels I, Huizenga H (2009) Activated region fitting: a robust high-power method for fMRI analysis using parameterized regions of activation. Human Brain Mapping 30: 2595–2605.

[111] Weeda W, Waldorp L, Grasman R, Van Gaal S, Huizenga H (2011) Functional connectivity analysis of fMRI data using parameterized regions-of-interest. NeuroImage 54: 410–416.

[112] Worsley K, Poline J, Friston K, Evans A (1997) Characterizing the response of PET and fMRI data using multivariate linear models. NeuroImage 6: 305–319.

[113] Xie X, Cao Z, Weng X, Jin D (2009) Estimating intrinsic dimensionality of fMRI dataset incorporating an AR(1) noise model with cubic spline interpolation. Neurocomputing 72: 1042–1055.

[114] Yue Y, Loh J, Lindquist M (2010) Adaptive spatial smoothing of fMRI images. Statistics and Its Interface 3: 3–13.

[115] Zhang C (2010) Statistical inference of minimum BD estimators and classifiers for varying-dimensional models. Journal of Multivariate Analysis 101: 1574 – 1593.

[116] Zhang C, Yu T (2008) Semiparametric detection of significant activation for brain fMRI. Annals of Statistics 36: 1693–1725.

[117] Zhang C, Lu Y, Johnstone T, Oakes T, Davidson R (2008) Efficient modeling and inference for event-related fMRI data. Computational Statistics & Data Analysis 52: 4859–4871.

[118] Zhang J, Tuo X, Yuan Z, Liao W, Chen H (2011) Analysis of fMRI Data Using an Integrated Principal Component Analysis and Supervised Affinity Propagation Clustering Approach. IEEE Transactions on Biomedical Engineering 58: 3184–3196.

[119] Zhang T, Li F, Beckes L, Brown C, Coan J (2012) Nonparametric inference of the hemodynamic response using multi-subject fMRI data. NeuroImage 63: 1754-1765.
